# Modulating Post-Stroke Inflammation with FDA-Approved Immunotherapies: A Literature Review

**DOI:** 10.3390/ijms27041700

**Published:** 2026-02-10

**Authors:** Eduardo Álvarez-Rivera, Pamela Rodríguez-Vega, Fabiola Colón-Santiago, Armeliz Romero-Ponce, Fabiola Umpierre-Lebrón, Paola Roig-Opio, Aitor González-Fernández, Tiffany Rosa-Arocho, Laura Santiago-Rodríguez, Ana Martínez-Torres, Gerson G. Santiago-González, James Llorens-Mercado, Jordan Acevedo-Rico, Victoria Bermúdez-Fosse, Naiara Hernández-Santisteban, Claudia Rodríguez-Castellanos, Carola García-Calderín, Fabyana Gómez-Irrizary, Solianne Martínez-Jiménez

**Affiliations:** 1 Microbiology and Immunology Department, Universidad Central del Caribe, Bayamón, PR 00960-6032, USA; eduardo.alvarez@uccaribe.edu; 2 School of Medicine, Universidad Central del Caribe, Bayamón, PR 00960-6032, USA; 3 Department of Science and Technology, Interamerican University, San Juan, PR 00919-1293, USAnaiarai.hernandezsan@intermetro.edu (N.H.-S.); 4 Department of Biology, University of Puerto Rico, San Juan, PR 00925-253, USA; 5 Neuroscience Department, Universidad Central del Caribe, Bayamón, PR 00960-6032, USA

**Keywords:** immunotherapy, stroke, inflammation, microglia, neuroinflammation, cytokines

## Abstract

Stroke has been a topic of extensive research due to its debilitating consequences and high mortality. New findings offer a deeper understanding of specific factors that affect post-stroke recovery and identify therapies that may facilitate this process. One such factor is post-stroke neuroinflammation, a complex and time-dependent process in which acute immune responses can cause significant secondary inflammatory damage if the process is prolonged. Microglia are neuronal immune cells that are highly reactive to cytokines in the neuroenvironment and can, in turn, affect the inflammatory cascades that originate after stroke, making them ideal candidates for immunomodulation in the brain. Many FDA-approved immunotherapies have been found to target distinct inflammatory signaling molecules and responders, including IL-6 inhibitors, IL-13 inhibitors, IL-12/IL-23 inhibitors, B-cell modulators, type I interferon inhibitors, CAR T-cell therapy, calcineurin inhibitors, complement inhibitors, and JAK-STAT pathway inhibitors. The FDA-approved immunotherapies discussed in this review demonstrate potential in modulating the immune response after stroke by targeting key inflammatory pathways involved in secondary brain injury. Future research should focus on defining optimal therapeutic windows, identifying suitable patient populations, determining the most appropriate timing of therapy, and targeting specific immune mechanisms to balance the attenuation of harmful inflammation with the preservation of reparative processes.

## 1. Introduction

A stroke is a cerebrovascular disease that results in damage to the brain. It is currently the second leading cause of death worldwide, as well as a major contributor to long-term disability. Approximately 85% of strokes are ischemic, with hemorrhagic strokes comprising the remaining 15%. In the United States, stroke is the fifth leading cause of morbidity and mortality, with up to 91% of risk factors being modifiable, including diabetes and hypertension [1]. Collectively, the high prevalence, morbidity, and long-term disability associated with stroke underscore its substantial public health burden and the need for therapeutic approaches that address both acute injury and downstream neurological consequences.

Ischemic stroke occurs due to the narrowing or occlusion of cerebral arteries, resulting in insufficient blood supply. This event triggers a rapid and complex inflammatory response characterized by the activation of immune cells and the release of inflammatory mediators [2]. Within minutes to hours of ischemic injury, hypoxia and tissue damage activate cerebral endothelial cells, leading to an increase in the expression of adhesion molecules, including ICAM-1, VCAM-1, and E-selectin. These processes compromise the integrity of the blood–brain barrier (BBB), facilitating infiltration of circulating immune cells. Simultaneously, resident microglia become activated and release pro-inflammatory cytokines (e.g., TNF-α, IL-1β), chemokines, and reactive oxygen species, creating an environment that recruits peripheral immune cells. Neutrophils are among the first leukocytes to infiltrate, typically within 24 h, contributing to tissue damage through the release of proteolytic enzymes (such as MMP-9) and reactive oxygen species, while also promoting microvascular obstruction [2,3].

Over the following days, monocytes infiltrate the brain and differentiate into macrophages, exhibiting both pro- and anti-inflammatory roles depending on the microenvironment. T lymphocytes, particularly CD4+ and CD8+ subsets, also infiltrate the ischemic brain, exacerbating injury through the release of cytokines and direct cytotoxicity. While the early inflammatory response is mainly detrimental, specific immune components in the subacute and chronic phases may support repair processes such as angiogenesis and tissue remodeling. Thus, post-stroke inflammation is a dynamic process that is both harmful and beneficial, representing potential therapeutic targets to modulate immune activity and improve neurological outcomes [3].

Recent transcriptomic studies have further highlighted that post-stroke immune responses are highly dynamic, with molecular signatures changing rapidly over time and differing between younger and older brains [4]. Microglial responses also vary according to anatomical location, vascular association, and temporal stage, underscoring the complexity and heterogeneity of post-stroke inflammation.

Importantly, prior clinical attempts to modulate post-stroke inflammation have produced mixed or negative results and, in some instances, worsened outcomes. Trials targeting leukocyte adhesion molecules (e.g., anti-ICAM-1), myelin-associated inhibitors, and selectins failed to demonstrate functional benefit and, in certain cases, increased mortality or adverse events. These findings demonstrate that post-stroke inflammation is neither uniform nor static, and that therapeutic efficacy depends on timing, cellular specificity, and patient-related factors such as age and baseline immune status [4]. Accordingly, the immunotherapies discussed in this review are not proposed as direct or universal stroke treatments but rather as mechanistically informative candidates to guide the development of more precise, targeted immunomodulatory strategies.

Given that many of these pathological processes are mediated by immune cell activation and cytokine signaling, interventions that modulate immune function may alter the extent of secondary brain injury after stroke. Immunotherapy, traditionally applied in oncology, has also been utilized for immunodeficiencies, autoimmune disorders, and inflammatory diseases. Thus, we investigate the potential for immune therapies to attenuate post-stroke inflammation, potentially improving rehabilitation outcomes. In this review, we examine FDA-approved immunotherapies from the past decade, focusing on their mechanisms of action and potential relevance for modulating post-stroke neuroinflammation.

## 2. Post-Stroke Neuroinflammation: A Look into the Brain’s Inflammatory Immune Responses

Post-stroke neuroinflammation is a complex and dynamic process involving coordinated interactions between resident central nervous system cells and infiltrating peripheral immune cells. This inflammatory network includes microglia, astrocytes, endothelial cells, and circulating leukocytes, regulated through cytokine and chemokine signaling. Among these components, microglia play a prominent role as early responders and integrators of inflammatory cues following ischemic injury.

Microglia, the resident immune cells of the CNS, are among the first responders to neuronal injury following stroke [5]. Consistent with their role, microglia rapidly respond to tissue damage following ischemic injury. Ischemic stroke leads to oxygen and nutrient deprivation, neuronal injury, cerebral edema, BBB breakdown, and a pronounced neuroinflammatory response. This environment triggers the release of damage-associated molecular pattern molecules (DAMPs), including extracellular ATP and UTP, which activate microglia [6].

Under physiological conditions, microglia perform immune surveillance and contribute to homeostatic regulation within the CNS [7]. However, the onset of stroke disrupts oxygen and nutrient supply, leading to neuronal injury, cerebral edema, breakdown of the blood–brain barrier (BBB), and a pronounced neuroinflammatory response. This environment leads to the release of damage-associated molecular pattern molecules (DAMPs), including extracellular ATP and UTP, which act as potent molecular triggers for microglial activation [7].

**Figure 1:**
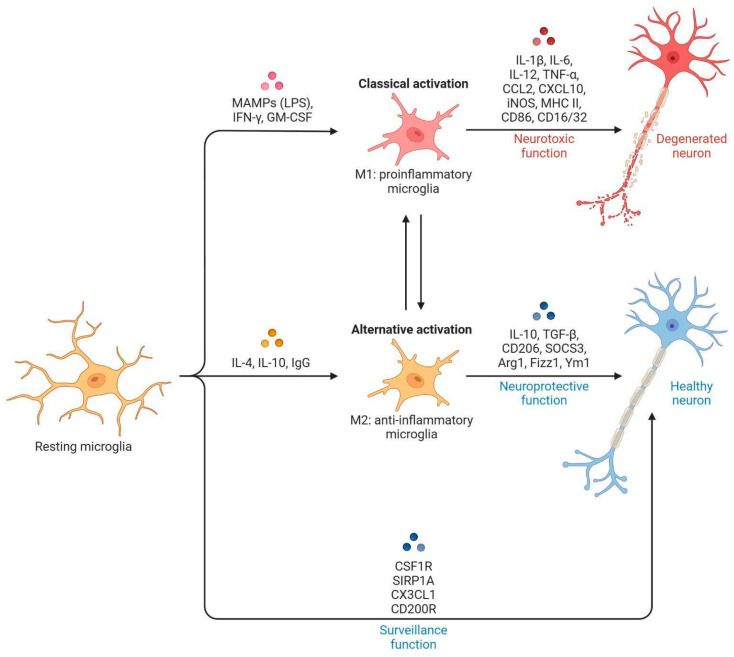
**Microglia activation**. Graphical summary of molecular signals associated with microglial activation. Created in BioRender. Martinez, S. (2025) https://BioRender.com/5n6uia6 (accessed on 30 December 2025).

Activated microglia exhibit distinct functional phenotypes, or polarization states, which modulate neuroinflammation (Figure 1). The M1 phenotype is pro-inflammatory, producing cytokines such as TNF-α, IL-1β, and IL-6, along with reactive oxygen species and inducible nitric oxide synthase (iNOS), contributing to neuronal damage. In contrast, the M2 phenotype is anti-inflammatory, promoting tissue repair through secretion of IL-10, TGF-β, IGF-1, GDNF, and BDNF, as well as phagocytosis of cellular debris [8,9]. The balance between these phenotypes plays a crucial role in determining whether post-stroke inflammation leads to progressive injury or supports recovery.

Microglial polarization is dynamic over time. M2-like microglia appear as early as 12 h post-insult but decline after 5–7 days, whereas M1 populations increase, peaking at day five. Persistent M1 activation exacerbates secondary neuronal injury and impedes recovery. Microglia also interact with astrocytes, neurons, and oligodendrocytes, influencing neuroinflammation and repair. Pro-inflammatory microglia can induce A1 astrocyte activation, oligodendrocyte damage, and BBB disruption, whereas M2 microglia support A2 astrocytes, oligodendrocyte differentiation, and BBB integrity [10].

Microglia engage in complex bidirectional communication with neurons, particularly in the context of ischemic injury. Neurons are among the most vulnerable cells during stroke and serve as both targets and modulators of the neuroimmune response [11]. They express a variety of regulatory cues commonly classified as “on” and “off” signals that influence microglial activation. For instance, excitotoxic neuronal injury, often mediated by excessive glutamate, can stimulate microglia to release pro-inflammatory cytokines and reactive oxygen species [12]. Neurons also interact directly with microglia via ligand–receptor pairs. Such interactions involve the chemokine fractalkine (CX3CL1) and its receptor, CX3CR1, which is predominantly expressed on microglia [7]. Under pro-inflammatory conditions, neurons increase the expression of CX3CL1, thereby enhancing neuroimmune signaling and microglial activation [7]. Furthermore, neurons may release “help-me” signals that promote microglial phagocytosis of neurotoxic debris and stimulate the production of antioxidant enzymes, thereby supporting neuronal survival [12].

Although microglial activation is often described using simplified phenotypic frameworks, increasing evidence highlights substantial heterogeneity among microglial populations depending on their anatomical location and microenvironment [7,8,9,10,11,12]. Microglia associated with the neurovascular unit, including vessel- and capillary-associated microglia, may exhibit distinct activation thresholds, temporal dynamics, and sensitivities to inflammatory cues compared to parenchymal microglia. These spatial and functional differences underscore the complexity of post-stroke neuroinflammation and suggest that immune-modulating therapies may exert variable effects across microglial subpopulations.

These findings position microglia as one of the central regulatory elements within the broader post-stroke neuroinflammatory network, interacting with other CNS-resident and peripheral immune cells. As key sensors and modulators of inflammatory signaling, microglia represent an important point of convergence for many immune pathways targeted by FDA-approved immunotherapies.

## 3. FDA-Approved Immunotherapies: Potential for Stroke Treatment

CD8+ T cells are among the first responders to enter the brain after an ischemic stroke, contributing to both acute inflammatory injury and longer-term immune regulation. Experimental studies suggest that specific CD8+ T-cell subsets may also exert neuroprotective effects, as reintroduction of CD8+ T cells after stroke in mice has been shown to reduce infarct size and improve long-term neurological recovery [13].

In a distinct clinical context, advances in immunotherapy have led to the FDA approval of chimeric antigen receptor (CAR) T-cell therapies, which involve ex vivo genetic modification of patient T cells to enhance immune activation against malignant targets. Although CAR T-cell therapies are not intended for stroke treatment, they are discussed here to highlight the profound consequences of excessive T-cell activation, including cytokine release and neurotoxicity, which are highly relevant considerations when evaluating immune-based strategies in the post-stroke brain.

ABECMA (idecabtagene vicleucel) is a CAR T-cell therapy used for patients with multiple myeloma, approved by the FDA in March 2021. This medication can only be used after the patient has not responded to proteasome inhibitors and anti-CD38 monoclonal antibodies. ABECMA binds to a protein called BCMA, which is found on the myeloma cells [14]. This medication can cause an immune effector cell-associated neurotoxicity syndrome. It can manifest as confusion, seizures, or cerebral edema [15]. Implementing this medication in the regimen of post-stroke patients, given these side effects, would not be beneficial. A more advanced form of CAR T-cell therapy should be developed.

Another CAR T-cell therapy is Tecartus (brexucabtagene autoleucel), which is used for patients suffering from B-cell malignancies. It was approved in October 2021; the main target is CD19, and it has two indications. The first is for adult patients with relapsed or refractory B-cell precursor acute lymphoblastic leukemia. The second indication is for relapsed or refractory mantle cell lymphoma. It does have a boxed warning for cytokine release and neurologic toxicities [16]. Similar to ABECMA, Tecartus is another immunotherapy agent that could be detrimental due to its side effects.

From another perspective, Anktiva (nogapendekin alfa inbakicept-pmln) is the first-in-class IL-15 receptor agonist for BCG-unresponsive non-muscle-invasive bladder cancer, approved by the FDA in April 2024. This medication sets a new standard for immunotherapies beyond checkpoint inhibitors. It works by activating the patient’s own natural killer cells and CD8+ killer T cells and simultaneously stimulates the proliferation of memory killer T cells. This new mechanism enables a durable complete response [17]. IL-15 is known to contribute to post-ischemic inflammation by promoting infiltration and activation of immune cells in the brain, which would be detrimental in the acute phase of stroke. Although Anktiva functions as a potent IL-15 receptor agonist and is beneficial in oncologic settings, its mechanism would probably exacerbate neuroinflammatory injury following ischemic stroke. However, these findings highlight the importance of IL-15 as a therapeutic target. Instead of intense and systemic activation, carefully timed and cell-specific IL-15 modulation may hold future neuroprotective potential. This would require careful consideration and further studies. Studying cytokine pathways, including those involving IL-15, is critical for developing effective methods to halt inflammatory cascades that can impede recovery after a stroke.

Along those lines, Sarilumab (Kevzara) is an interleukin-6 receptor antagonist that suppresses downstream pro-inflammatory pathways. Even though IL-6 blockade is well established in chronic systemic inflammatory diseases, like rheumatoid arthritis, IL-6 also plays a central role in the acute inflammatory response after an ischemic stroke. Following cerebral ischemia, IL-6 levels rise in the brain and peripheral circulation, stimulating leukocyte recruitment, blood–brain barrier disruption, and amplification of cytokine cascades that further cause secondary neuronal injury. Since elevated IL-6 concentrations have been associated with larger infarct volumes and worse functional outcomes, it could be a potential biomarker and therapeutic target. By reducing IL-6-mediated signaling, sarilumab may help modulate post-stroke inflammation, potentially limiting secondary tissue damage and improving neurological recovery [18].

On a similar note, satralizumab (Enspryng) is a monoclonal antibody designed to reduce inflammation by targeting the interleukin-6 (IL-6) receptor. By blocking the IL-6 receptor, satralizumab helps to calm this immune response, preventing immune cells from causing further damage to the nervous system. It is currently FDA-approved for neuromyelitis optica spectrum disorder (NMOSD). By targeting this specific cytokine pathway, satralizumab has shown the ability to reduce relapses and preserve neurological function [19]. Given its impact on immune signaling and neuroinflammation, like sarilumab, satralizumab may also hold promise in post-stroke inflammation, where IL-6 contributes to secondary injury.

Ebglyss (lebrikizumab) is an interleukin-13 (IL-13) antagonist, preventing the interaction with its receptor by binding directly to IL-13. Although IL-13 is best known for its role in type 2 inflammatory diseases, such as atopic dermatitis, it also has an immune-regulating role in areas outside of allergic tissues [20,21]. IL-13 influences macrophage polarization, microglial activation, and cytokine balance. These processes are highly relevant in the neuroinflammatory cascade after an ischemic stroke. As previously stated, the dysregulated immune response that happens after a stroke contributes to secondary brain injury. Emerging evidence suggests that IL-13 signaling may participate in modulating post-stroke inflammation, potentially affecting the balance between damaging pro-inflammatory activity and reparative immune pathways. Thus, targeted IL-13 inhibition with ebglyss warrants investigation as a strategy to modulate neuroinflammation and limit secondary injury after stroke [22].

Furthermore, Wezlana (ustekinumab-auub) is a biosimilar to ustekinumab, a monoclonal antibody that targets the p40 subunit shared by interleukins-12 (IL-12) and 23 (IL-23), thereby inhibiting their downstream inflammatory signaling. While IL-12 and IL-23 are classically associated with systemic immune-mediated diseases, they also play important roles in shaping neuroinflammatory responses. Both cytokines influence T-cell differentiation and activation, promoting Th1 and Th17 pathways that have been implicated in post-ischemic immune activation in the central nervous system. After a stroke, infiltration of peripheral immune cells and activation of resident microglia contribute to secondary injury through sustained cytokine production and cytotoxic inflammation. Dysregulation of IL-12 and IL-23-driven pathways may intensify these processes, connecting peripheral immune signaling to ongoing neuronal damage. By modulating these upstream cytokine networks, ustekinumab biosimilars, such as Wezlana, may represent a potential strategy for reducing harmful immune responses and limiting secondary neuroinflammation after stroke [23,24].

Belimumab (Benlysta) is a monoclonal antibody approved by the FDA in 2011 for treating systemic lupus erythematosus (SLE) and subsequently for lupus nephritis (LN). It functions by inhibiting the B lymphocyte stimulator (BLyS), thereby reducing B-cell activity, which is pivotal in the pathogenesis of lupus [25]. As belimumab can modulate inflammatory markers, it may be beneficial in managing post-stroke inflammation. Acute inflammation is a key factor in stroke recovery, and by reducing inflammatory responses, belimumab may enhance healing processes in the brain. Notably, research indicates that persistent inflammation can exacerbate kidney damage in lupus patients. [26] Thus, belimumab’s ability to lower inflammatory markers is crucial not only for renal health but also for overall recovery after inflammatory events, such as a stroke.

Ocrelizumab (Ocrevus) is a monoclonal antibody that targets CD20-positive B cells, a specific subset of immune cells involved in the chronic inflammation seen in multiple sclerosis (MS). B cells contribute to the inflammatory cascade by producing pro-inflammatory cytokines, presenting antigens, and helping to activate T cells, which can then attack the central nervous system. Ocrelizumab’s ability to modulate CNS-directed immune responses makes it not only a breakthrough for MS but also highlights its potential in other CNS inflammatory conditions, such as neuroinflammatory responses after stroke, where B-cell-driven pathways may contribute to prolonged inflammation and injury [27]. Likewise, Anifrolumab, known by the brand name Saphnelo, is a monoclonal antibody approved in 2021 for the treatment of moderate to severe systemic lupus erythematosus. It works by targeting the type I interferon receptor, which is instrumental in driving the inflammatory processes associated with lupus. By inhibiting this receptor, anifrolumab reduces the activity of type I interferons, key players in autoimmunity and inflammation [28]. Clinical studies have shown that this medication can meaningfully decrease disease activity, offering a valuable option for patients who have not responded well to traditional therapies. By managing inflammation, anifrolumab may potentially have implications for preventing secondary damage and promoting recovery after a stroke based on the overlap of mechanisms of inflammatory pathways.

Natalizumab (Tysabri) is a monoclonal antibody that works by targeting α4-integrin, a molecule found on the surface of white blood cells. Under normal inflammatory conditions, these integrins help immune cells adhere to blood vessel walls and migrate into tissues, including the central nervous system (CNS). In diseases like multiple sclerosis (MS), this process allows harmful immune cells to cross the blood–brain barrier and attack healthy brain and spinal cord tissue. By blocking α4-integrin, Tysabri prevents this infiltration, thereby reducing neuroinflammation and slowing disease progression [29]. Its mechanism directly interrupts a step in the inflammatory cascade: leukocyte migration. This not only makes it highly effective in treating relapsing forms of MS but also suggests potential therapeutic value in other CNS conditions characterized by immune cell infiltration, such as post-stroke inflammation, where blood–brain barrier disruption and leukocyte entry can exacerbate injury. By keeping immune cells out of the CNS, natalizumab offers a powerful approach to protecting neural tissue from immune-mediated damage.

Alternatively, voclosporin (Lupkynis) is a calcineurin inhibitor approved for the treatment of lupus nephritis. It works by suppressing T-cell activation through inhibition of calcineurin signaling [30]. Although its current clinical use is focused on lupus nephritis, this mechanism may also be relevant in the context of post-stroke neuroinflammation. By diminishing T-cell activation and downstream inflammatory signaling, voclosporin could, in theory, help limit immune-mediated neuronal injury since T-cell infiltration after a stroke contributes to secondary damage. Experimental studies suggest that calcineurin signaling plays a role in post-stroke inflammatory pathways, providing a mechanistic rationale for considering calcineurin inhibitors in this setting [31]. Direct evidence supporting the use of voclosporin after stroke is still limited, and further research is needed to better understand its effects on neuroimmune interactions and functional recovery.

Tavneos (avacopan) is an FDA-approved oral medication that acts as a selective antagonist of the complement 5a receptor (C5aR), a key participant in the innate immune response. C5a is one of the most potent pro-inflammatory components of the complement cascade, known for activating and recruiting neutrophils to sites of tissue injury. By blocking C5aR, avacopan reduces neutrophil migration, activation, and the release of damaging enzymes and reactive oxygen species, all of which contribute to inflammation and tissue destruction in diseases like ANCA-associated vasculitis [32]. Although its current FDA approval is limited to systemic vasculitis, this mechanism directly targets a critical step in the inflammatory cascade that is also involved in CNS pathology. In conditions such as ischemic stroke, excessive neutrophil and complement activity can damage the blood–brain barrier and exacerbate neural injury. Therefore, avacopan’s ability to suppress neutrophil-driven inflammation suggests potential therapeutic implications in ischemic stroke.

Another promising strategy is to disrupt the JAK-STAT signaling pathway, a key driver of pro-inflammatory cytokine activity. Upadacitinib (Rinvoq) is a selective Janus kinase 1 (JAK1) inhibitor that reduces the production and signaling of various cytokines, leading to improved disease activity scores and enhanced quality of life across multiple inflammatory conditions. Since its FDA approval in August 2019 for rheumatoid arthritis, it has received additional approvals for psoriatic arthritis, atopic dermatitis, ulcerative colitis, Crohn’s disease, ankylosing spondylitis, and non-radiographic axial spondyloarthritis [33,34]. Given the involvement of JAK-STAT signaling in neuroinflammation, its mechanism of action raises the possibility that JAK1 inhibition could be relevant in the context of post-stroke inflammatory responses [21].

Baricitinib (Olumiant), an oral inhibitor of JAK1 and JAK2, was first approved in May 2018 for the treatment of rheumatoid arthritis. It later received Emergency Use Authorization during the COVID-19 pandemic and gained full FDA approval in May 2022 for the treatment of hospitalized adults who require oxygen therapy. Additionally, it was approved for the treatment of alopecia areata in June 2022. Through its anti-inflammatory effects, baricitinib has been shown to reduce disease activity in rheumatoid arthritis, lower mortality and accelerate recovery in COVID-19, and promote hair regrowth in alopecia areata. Although it has not been studied in the context of stroke, its broad effects on systemic immune pathways raise the theoretical possibility that it could influence inflammatory mechanisms following cerebrovascular events [35,36].

## 4. Discussion

The outlook for stroke recovery is becoming increasingly optimistic as emerging research brings forward more personalized and effective therapeutic strategies. Several FDA-approved monoclonal antibodies target pro-inflammatory cytokines and immune signaling pathways, suggesting potential translational relevance for post-stroke inflammation (Table 1). Immunomodulation, particularly through enhancement of regulatory T-cell responses, has shown considerable promise in reducing post-stroke inflammation and facilitating neural repair [37]. Additionally, precise manipulation of microglial activation states may help limit secondary neuronal injury and enhance neuroplasticity, highlighting the central role of the immune microenvironment in recovery trajectories [38].

However, previous clinical attempts to modulate post-stroke inflammation have yielded mixed or negative results. Trials targeting leukocyte adhesion molecules (e.g., anti-ICAM-1), myelin-associated inhibitors, and selectins failed to improve outcomes and, in some cases, increased adverse events or mortality [4]. These experiences underscore that post-stroke inflammation is neither uniform nor static and that therapeutic efficacy depends critically on timing, cellular specificity, and patient factors such as age and baseline inflammatory state. Accordingly, the immunotherapies discussed here are intended as mechanistically informative candidates, rather than direct stroke treatments, to guide more precise future strategies [4,39].

In parallel, precision medicine approaches integrated with artificial intelligence are improving the ability to predict patient-specific outcomes by synthesizing data from neuroimaging, electrophysiological monitoring, and clinical profiles. These predictive models enable the development of more personalized interventions that could optimize functional recovery for each stroke patient. Together, these interdisciplinary innovations are shifting stroke care from a reactive, generalized model to an individualized and precision-guided restoration. In addition, future research should focus on precise patient stratification, identifying stage-specific therapeutic windows, and developing biomarker-guided approaches capable of limiting harmful inflammation while preserving reparative immune functions [4,39].

### 4.1. Monoclonal Antibodies and Cytokine Modulators

- IL-6 inhibitors: Sarilumab (Kevzara) and satralizumab (Enspryng) block the interleukin-6 receptor, thereby dampening the pro-inflammatory response [18,19].
- IL-13 inhibitor: Lebrikizumab (Ebglyss) antagonizes IL-13, reducing inflammatory signaling [20,21].
- IL-12/IL-23 inhibitors: Ustekinumab (Wezlana) targets the p40 subunit shared by IL-12 and IL-23, pathways implicated in neuroinflammation [23,24].
- B-cell modulators: Belimumab (Benlysta) reduces B-cell activity by inhibiting the B lymphocyte stimulator (BLyS), while ocrelizumab (Ocrevus) targets CD20-positive B cells [25,26,27].
- Type I interferon inhibitors: Anifrolumab (Saphnelo) blocks type I interferon signaling, reducing systemic inflammation [28].

### 4.2. Other Immunomodulatory Agents

- Calcineurin inhibitors: Voclosporin (Lupkynis) suppresses T-cell activation and proliferation [30].
- Complement inhibitors: Avacopan (Tavneos) antagonizes C5a receptor signaling, reducing neutrophil-mediated tissue damage [32].
- JAK-STAT pathway inhibitors: Upadacitinib (Rinvoq) and baricitinib (Olumiant) reduce pro-inflammatory cytokine activity [34,35,40,41].
- Immune cell-based therapies: ABECMA and Tecartus use genetically modified T cells to target malignant cells and potentially harness T-cell modulation for neuroprotection [15,42,43].

While FDA-approved immunotherapies target inflammatory pathways relevant to post-stroke pathology, their translation into effective stroke treatments presents significant biological and clinical challenges. Post-stroke inflammation is highly dynamic and temporally regulated, with immune responses that may be deleterious in the acute phase yet supportive of tissue repair during subacute and chronic stages. Consequently, indiscriminate or poorly timed immunomodulation may disrupt beneficial reparative processes, partially explaining why several prior anti-inflammatory strategies have failed to improve clinical outcomes in stroke populations.

Another major translational challenge lies in the systemic nature of many FDA-approved immunotherapies. Agents that modulate cytokine signaling, lymphocyte activation, or complement pathways exert broad immunological effects that extend beyond the central nervous system. In the context of stroke, where patients are already vulnerable to infections and secondary complications, systemic immunosuppression represents a significant safety concern. Thus, therapeutic success may depend on careful patient stratification, dose optimization, and selection of treatment windows that minimize systemic risk while attenuating harmful neuroinflammation.

Importantly, many of the immunotherapies discussed in this review may influence post-stroke neuroinflammation indirectly rather than by targeting a single cell type. Cytokine inhibitors, JAK-STAT pathway modulators, and B- or T-cell-directed therapies can alter peripheral immune activation, leukocyte trafficking, and circulating inflammatory mediators, which in turn shape microglial activation and blood–brain barrier integrity. From this perspective, immunomodulation may function as a network-level intervention, affecting interconnected inflammatory pathways rather than isolated molecular targets. This systems-level view supports the exploration of coordinated or sequential immunomodulatory strategies tailored to specific inflammatory phases of stroke.

Future translational efforts may therefore benefit from integrative approaches that combine immune profiling, neuroimaging, and clinical parameters to identify patients most likely to benefit from targeted immunotherapy. Rather than relying on single-agent interventions, combination or stage-specific strategies may offer greater potential to balance suppression of harmful inflammation with preservation of immune-mediated repair mechanisms. Such approaches remain hypothesis-generating and require rigorous clinical investigation, but they underscore the need for precision-guided immunomodulation in post-stroke care.

Overall, FDA-approved immunotherapies demonstrate significant potential in modulating the immune response after stroke by targeting key inflammatory pathways involved in secondary brain injury. Therapies directed at cytokine signaling, B-cell activity, or T-cell function may reduce neuroinflammation, preserve BBB integrity, and promote neuroprotection. Cell-based approaches, though promising, require careful consideration due to neurotoxicity risks. Collectively, these observations support the translational potential of immunomodulation in neuroinflammatory conditions and provide a foundation for future clinical investigation.

## 5. Challenges and Future Directions

Despite promising preclinical results, translating immunotherapies to clinical stroke care faces challenges. Timing is critical; early administration may reduce secondary injury, but late administration could interfere with tissue repair. Safety is another concern, as systemic immunosuppression may increase the risk of infection.

Future research should focus on identifying optimal therapeutic windows, patient stratification based on inflammatory profiles, and combination therapies targeting multiple immune pathways. Precision medicine approaches, including biomarker-guided interventions, may help optimize therapeutic efficacy while minimizing adverse effects.

## 6. Conclusions

This review highlights the central role of immune-mediated mechanisms in shaping post-stroke neuroinflammation and recovery, with a particular emphasis on microglial activation, cytokine signaling, and adaptive immune responses. Together, these processes contribute to secondary brain injury and represent points at which immune modulation may alter neurological outcomes after stroke.

Several FDA-approved immunotherapies originally developed for oncologic, autoimmune, and inflammatory conditions target molecular pathways implicated in post-stroke inflammation and therefore warrant consideration for therapeutic repurposing. Agents that modulate cytokine activity, T-cell responses, or B-cell function demonstrate mechanistic relevance in this context. However, immune-based therapies differ substantially in their safety profiles, and approaches associated with neurological toxicity, particularly specific cell-based therapies, require careful evaluation before application in stroke populations.

Overall, immunomodulation offers a promising strategy for addressing post-stroke inflammation beyond conventional reperfusion therapies, but rigorously designed clinical studies informed by molecular and transcriptomic insights are essential to determine whether these interventions can safely and meaningfully improve long-term neurological outcomes [4,38]. Future research should focus on defining optimal therapeutic windows, appropriate patient populations, therapeutic timings, and immune targets to balance attenuation of harmful inflammation with preservation of reparative processes. Careful clinical investigation will be essential to determine whether repurposed, FDA-approved immunotherapies can safely contribute to improved recovery and long-term neurological outcomes following stroke.

## Figures and Tables

**Table 1:** **Immunotherapies review summary**. Current FDA-approved immunotherapies target several signaling molecules implicated in the stroke inflammatory cascade, highlighting their therapeutic potential and need for further study.

Immunotherapy	Main Signaling Target	Approved Treatment
ABECMA	BCMA	Myeloma
Tecartus	CD19	B-cell malignancies
Anktiva	IL-15	Bladder cancer
Kevzara	IL-6	Rheumatoid arthritis
Enspryng	IL-6	NMOSD
Ebglyss	IL-13	Atopic dermatitis
Wezlana	IL-12, IL-23	Plaque psoriasis, psoriatic arthritis, Crohn’s disease, ulcerative colitis
Benlysta	BLyS	Systemic lupus erythematosus
Ocrevus	CD20+ B cells	Multiple sclerosis
Tysabri	α4-integrin	Multiple sclerosis
Lupkynis	Calcineurin	Lupus nephritis
Tavneos	C5aR	Systemic vasculitis
Rinvoq	JAK1	Rheumatoid arthritis
Oluminant	JAK1, JAK2	COVID-19, alopecia areata, rheumatoid arthritis

## Data Availability

No new data were created or analyzed in this study. Data sharing is not applicable to this article.

## Abbreviations

The following abbreviations are used in this manuscript:

FDA	Food and Drug Administration
IL	Interleukin
CAR	Chimeric antigen receptor
JAK-STAT	Janus kinase/signal transducers and activators of transcription
ICAM	Intercellular adhesion molecule
VCAM	Vascular cell adhesion molecule
BBB	Blood–brain barrier
TNF-α	Tumor necrosis factor alpha
MMP-9	Matrix netalloproteinase-9
CD4	Cluster of differentiation 4
CD8	Cluster of differentiation 8
CNS	Central nervous system
DAMPs	Damage-associated molecular patterns
ATP	Adenosine triphosphate
UTP	Uridine triphosphate
iNOS	Inducible nitric oxide synthase
IGF-1	Insulin-like growth factor 1
TGF-β	Tumor growth factor beta
GDNF	Glial cell line-derived neurotrophic factor
BDNF	Brain-derived neurotrophic factor
CX3CL1	C-X3-C motif chemokine ligand 1
CX3CR1	C-X3-C motif chemokine receptor 1
TRL	Toll-like receptor
BCMA	B-cell maturation antigen
BCG	Bacillus Calmette–Guérin
NMOSD	Neuromyelitis optica spectrum disorder
Th2	T helper type 2 cell
SLE	Systemic lupus erythematosus
LN	Lupus nephritis
BLyS	B lymphocyte stimulator
PERR	Primary efficacy renal response
MS	Multiple sclerosis
C5aR	Complement 5a receptor
ANCA	Anti-neutrophil cytoplasmic antibody
